# Supplementary material for: Analysis of PPARγ Signaling Activity in Psoriasis
Source: Int J Mol Sci. 2021 Aug 10;22(16):8603. doi: 10.3390/ijms22168603 (PMC8395241; doi:10.3390/ijms22168603)
Supplement: Supplementary file 1 [file ijms-22-08603-s001.zip › Supplemental materials_Analysis of PPARg signaling activity in psoriasis/Pathway models/Models images and html files/PPARG inhibits phenotypes assotiated with psoriasis/244917.html]

vascular remodeling


# Disease vascular remodeling

|  |  |
| --- | --- |
| URN | urn:agi-disease:vascular%20remodeling |
| Total Entities | 0 |
| Connectivity | 3558 |
| Name | vascular remodeling |

---

|  |  |
| --- | --- |
| Pathway | ROS in Angiotensin Mediated Cardiovascular Remodeling and Hypertrophy |
|  | PPARG inhibits phenotypes assotiated with psoriasis |
|  | efects selected |
|  | Neighbors of vascular remodeling |

---

|  |  |
| --- | --- |
| MedScan ID | 9017232 |

---

|  |  |
| --- | --- |
| Alias | vascular changes |
|  | changes of vascular |
|  | vascular remodeling |

---
